# Wireless Geophone Networks for Land Seismic Data Acquisition: A Survey, Tutorial and Performance Evaluation

**DOI:** 10.3390/s21155171

**Published:** 2021-07-30

**Authors:** Aliyu Makama, Koojana Kuladinithi, Andreas Timm-Giel

**Affiliations:** Institute of Communication Networks, Hamburg University of Technology, 21073 Hamburg, Germany; koojana.kuladinithi@tuhh.de (K.K.); timm-giel@tuhh.de (A.T.-G.)

**Keywords:** central control unit (CCU), geophones, seismic survey, wireless seismic data acquisition (WSDA), wireless geophone network (WGN)

## Abstract

Seismic data acquisition in oil and gas exploration employs a large-scale network of geophone sensors deployed in thousands across a survey field. A central control unit acquires and processes measured data from geophones to come up with an image of the earth’s subterranean structure to locate oil and gas traps. Conventional seismic acquisition systems rely on cables to connect each sensor. Although cable-based systems are reliable, the sheer amount of cable required is tremendous, causing complications in survey logistics as well as survey downtime. The need for a cable-free seismic data acquisition system has attracted much attention from contractors, exploration companies, and researchers to lay out the enabling wireless technology and architecture in seismic explorations. This paper gives a general overview of land seismic data acquisition and also presents a current and retrospective review of the state-of-the-art wireless seismic data acquisition systems. Furthermore, a simulation-based performance evaluation of real-time, small-scale wireless geophone subnetwork is carried out using the IEEE 802.11 g technology based on the concept of seismic data acquisition during the geophone listen or recording period. In addition, we investigate an optimal number of seismic samples that could be sent by each geophone during this period.

## 1. Introduction

A seismic survey is a method of obtaining the graphical representation of the earth’s subsurface structure by analysis of seismic waves. It has a vast area of applications, such as volcanic monitoring [1], earthquake early warning system [2], landslide monitoring [3], mineral resources survey [4], imaging of glaciers and ice sheets to monitor how change in climate affects the subglacial environment [5,6], etc. It is often achieved using a technique called “Seismic Reflection”. For almost a century, exploration companies commonly employ this method to determine ideal places to drill for oil and gas, as well as monitor and plan enhanced recovery programs [7]. A seismic survey can either be onshore or offshore. In an onshore survey, a network of sensors called “geophones” are deployed on the survey area, typically in a line or in a rectangular grid or other geometry, according to pre-defined survey parameters. The traditional approach of deploying these networks is to connect each sensor via cable. Cable-based surveys are known to have a lot of disadvantages, such as excess weight, reliability issues, complexities in deployment and maintenance, human resource costs [8], and other operational costs. According to [9], cables account for up to 50% of operational costs and 75% of the total equipment weight in land seismic surveys. Moreover, cables are prone to damages by natural and cultural causes, resulting in survey down-time as considerable field time is expended troubleshooting faulty cables [10]. The growing demand for better quality, high-density subsurface imaging is impelling seismic exploration companies to provide more recording channels in surveys. Future seismic surveys might require recording channels ranging from hundreds of thousands and above [11]. This will result to even more complex, cumbersome, and expensive logistics around cable-based exploration. To address the problems associated with cables, wireless seismic data acquisition systems have been proposed in recent years. The system utilizes seismic sensor nodes equipped with wireless transceivers to form a network of wireless geophone sensors that employ radio frequency communication technology. Although proposals for wireless acquisition systems date back to few decades ago [12], recent advancement in wireless technology may pave the way for a complete cable-free wireless seismic data acquisition system. Wireless Geophone Network (WGN) is the acronym that will be used to denote the network of geophones, describing a cable-free seismic data acquisition system. The goal of this paper is three-fold: First, we present a general overview of an onshore seismic survey in oil and gas exploration, underlying the acquisition methodology and requirements. Secondly, the “state-of-the-art” application of WGN in high-density seismic data acquisition and fundamental requirements necessary to set up these networks is outlined. Finally, we considered a subnetwork of wireless geophones in a seismic survey setting, with a focus on the data delivery stage of the survey, and evaluated the network performance based on the geophone-recording period to investigate the optimal number of seismic samples to be transmitted during this period.

The rest of the paper is organised as follows: Section 2 gives a general review on the fundamentals of a land seismic survey. Section 3 outlines the basic network requirements for onshore wireless seismic data acquisition (WSDA) systems. Section 4 gives an overview on the state-of-the-art wireless seismic exploration. In Section 5, we propose a WGN architecture and a recording period acquisition technique and, using a simulation, investigate the optimal number of seismic samples to be transmitted by each geophone. Section 6 presents the result analysis, followed by discussions of the investigation carried out and conclusion in Section 7 and Section 8, respectively.

## 2. Overview of Land Seismic Survey

**Figure 1:**
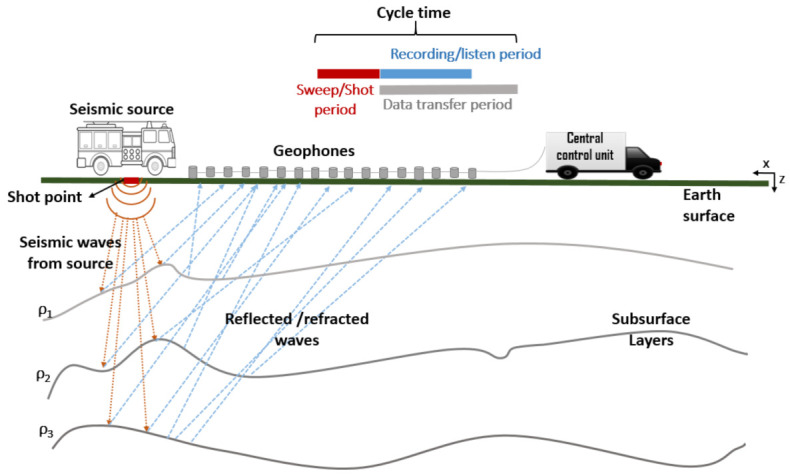
An onshore seismic survey [14].

The propagation of elastic waves through the earth subsurface is the basis of seismic exploration. Body waves that are capable of travelling through all distance levels of the earth’s subsurface and Interface waves that found near boundary of layers are the two classes of elastic waves. Most seismic surveys are based on the analysis of body wave signals produced by a seismic source and recorded at the earth’s surface [13], as illustrated in Figure 1. Shear or Secondary wave (S-wave) and Compressional or Primary waves (P-wave) are the two types of body waves that exhibit particle motion orthogonal and parallel to the direction of wave propagation, respectively. Using the wave travel velocity and the time required for the wave to return to the surface, the depth of different geological layers could be determined. The wave velocity value carries information on the type of rock or sediment in the subsurface. Depending on the subsurface layer’s density and elasticity, the velocities of these waves are expressed using Equations (1) and (2) below:

(1) $$V_{s}=\sqrt{\frac{\mu }{\rho }}$$

(2) $$V_{p}=\sqrt{\frac{k+(4\mu /3)}{\rho }}$$

where *k* is the compressional modulus, µ is the shear modulus, is the density of the subsoil, and $V_{s}$ and $V_{p}$ are the velocities of the secondary and primary waves, respectively.

In sedimentary materials, the elasticity and density strongly depends on the porosity of the layer. S-wave velocities (in the range of 100–500 m/s) are much slower in comparison with P-wave velocities, as such are termed secondary waves as it arrives later than the P-wave. P-wave velocities ranges from 200 to 800 m/s and 1500–2500 m/s for dry and water logged materials, respectively, for sediments such as clay, sand, or gravel. Similar to sound waves, these waves are reflected and refracted as they hit boundaries between different subsurface layers. The magnitude of the difference between boundary densities and seismic velocities defines the intensity of the reflected wave. The seismic wave energy is proportional to the square of its amplitude, and the propagation velocity is the product of its wavelength (λ) and frequency (*f*) [14].

The unavoidable exigency for oil and gas as the main source of energy and other numerous benefits and applications in everyday life has pushed exploration companies to heavily invest in the search for new reservoirs and technologies that will improve the quality of subterranean imaging and efficiency in oil exploration [9]. With the potential of much higher data resolution due to better sensors, seismic methods continue to gain attention for use in mineral exploration applications as compared to other traditional techniques used, such as magnetics, gravity, resistivity, and electro magnetics [15].

In an onshore survey, an energy source (explosives, thumper, vibroseis, sledgehammer, etc.) placed at a shot point generates a low-frequency seismic wave that propagates into the earth’s subsurface through various paths. These seismic waves are reflected and refracted as they experience changes in the geological layering of the earth. A network of seismic sensors or receivers (geophones) deployed in the survey area at the surface detects and records the reflections arriving at the surface during the period of time called “*geophone recording or listen period*” and converts them into electrical signals, which are then amplified, filtered, digitised, multiplexed, and sent down to the central control unit (CCU) for further processing to obtain a visual image of the earths subsurface structure [16], after which the source is moved to the next shot point, and the next record is taken. The acquired image is then interpreted by geophysicists or geologists to identify pockets of oil and gas before any test drilling commences. Figure 1 gives an illustration of the seismic survey process.

### 2.1. Survey Equipments

#### 2.1.1. Energy Source

Seismic energy sources can either be impulsive or vibratory.

- Impulsive: Explosives such as dynamite or ammonium nitrate are stuffed at the bottom of drilled holes on the survey field prior to the commencement of the data acquisition. The instrument operator starts a sequence of events that causes the explosives to detonate. Energy produced by the explosive is transmitted as a seismic wave signal that radiates outward in all directions via the earth subsurface, and reflections are recorded at the receivers on the surface [17].
- Vibratory: Vibrators (vibroseis) are the most commonly used sources in land seismic exploration [18] due to some advantages it has over impulsive sources, such as limited frequency band, low-power source, longer energy emission time [19], less safety concerns, as well as better control of sweep repetition. A vibrator is a vehicle-mounted energy source that converts an electrical signal into high-pressure hydraulic flow to vibrate and control a heavy base plate held in contact with the ground by the weight of the vibrator vehicle and isolated by an air-bag suspension [19]. A reference sweep signal encoded in the vibrator electronics is sent into the ground through the vibrator plates for a given period of time known as the *“sweep or shot time”*. Geophones at the surface records the data or seismic reflections for a duration called the *listen time* [20]. The period from the completion of the sweep to the end of recording is the listen time. The vibrator truck lifts the plates and then moves to the next shot point in the survey area. The procedure is repeated throughout the survey area. Vibrators have a typical signal frequency range of 5–511 Hz and a sweep length of up to 31 s [17]. One major drawback of vibrators is that they cannot be used in complex terrains, such as mountainous or marshy areas, or in jungles.

#### 2.1.2. Seismic Sensor

Seismic sensors used in an onshore seismic exploration can either be geophones or accelerometers. The most commonly used sensors are the geophones [21], which measure ground motion or velocity when shaken by an energy source and converts it into electrical energy. The moving-coil system design is typically employed in geophones [22]. It consists of a coil suspended by means of a spring and permanent magnet surrounding the coil. The motion of the ground causes the coil to move through the magnet’s field, thereby producing a voltage that is proportional to the relative ground vibration or the velocity of the coil. Basically, the sensitivity of the geophone is such that a unit ground velocity produces voltage measured in millivolt per inch per second (mV/ips) [17]. The geophone is a damped resonant system with a damping factor usually in the range of 0.5–0.7 and a resonant frequency usually from as low as 8 Hz to greater than 100 Hz, which provides adequate coupling for most seismic applications [21]. Waves travelling through the earth’s surface comprise of three orthogonal components in nature [23]. A normal geophone measures only the vertical component of the ground’s motion velocity [21]. A three-component (3-C) geophone, with three spring-mounted coils arranged orthogonally in a single case, measures the three mutually orthogonal components of the particle velocity [23].

**Figure 2:**
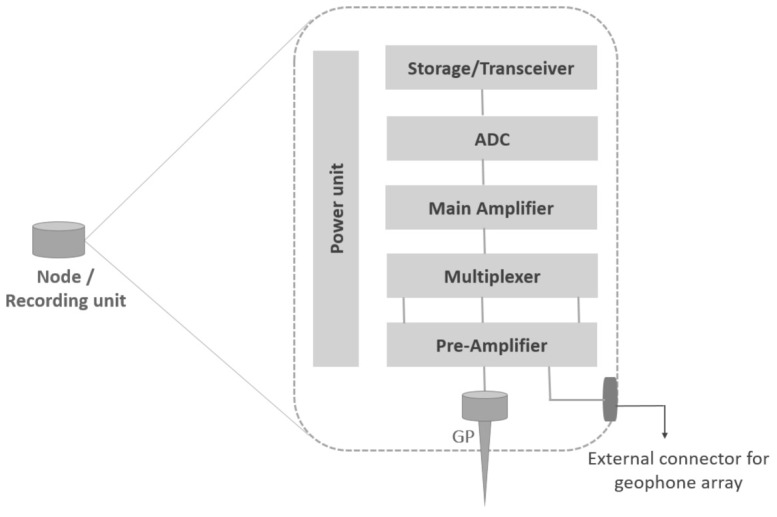
The components of a node or recording unit [16].

Seismic surveys on land are often conducted by deploying an array of geophones coupled together in a geometric pattern to a single unit called the “acquisition” or “recording” unit to enhance the weakly reflected signal level and also reduce the coherent noise level [16] over the survey area. Advancements in modern electronics have given rise to completely cable-free single seismic recording unit referred to as “node”. A node is a stand-alone acquisition unit, which is comprised of batteries, a processing unit, analogue-to-digital converter (ADC), filters, memory, clock for timing precision, transceivers, internal geophone sensor (GP), as well as an external connector for coupling array of geophones [10], as shown in Figure 2. Throughout the rest of this paper, we use the term “geophone” to denote a stand-alone wireless node or recording unit comprising of a single internal geophone.

Reflected signals from geophones pass through an amplifier and ADC for signal amplification and digitization, respectively, after which it is transmitted to the CCU for further processing. The CCU is also responsible for monitoring geophones as well as timing during the survey. A stream of digitised samples drawn from a single geophone is called a “seismic channel” [9]. The rate at which data is generated (*D*) from one seismic channel depends on the sampling interval ($T_{s}$) with which the signal of the reflected wave is sampled and the resolution (*R*) of the ADC used. Depending on the seismic objective, instrument used, and required bandwidth, the sampling interval typically ranges from 0.25 ms to 8 ms. The lower the sample interval, the higher the frequency range that can be recorded. Above the Nyquist frequency, frequencies are sampled incorrectly. Similarly, the larger the number of bits in the ADC the greater the dynamic range, i.e., the range of signal amplitudes that can be converted without distortion [16]. Equation (3) gives the relationship between *D*, *R*, and $T_{s}$.

(3) $$D=\frac{R}{T_{s}}$$

For a sampling time of 1 ms, a geophone with a 24 ADC resolution will generate data at a rate of 24 kbit/s. Three times as much data rate will be generated with a 3-C geophone. A geophone with *S* seismic channels will generate an aggregate data stream of S×D. Employing data compression techniques will reduce the amount of data generated, which consequently leads to shorter data transmission time [24], as well as decrease in network bandwidth costs.

### 2.2. Real Time vs. Blind Data Acquisition

Seismic data acquired by a geophone for each sweep can be retrieved at the CCU in either of the following ways: (i) stored internally and retrieved later at the CCU at the end of the survey or (ii) transmitted instantly to the CCU [25]. The acquisition system is referred to as blind when geophones have no means of transmitting data (i.e., equipment quality control data, noise level monitoring, or seismic data) during the survey to the CCU for monitoring. The equipment quality control (QC) data transmitted could be the status of either the battery level, GPS, storage capacity, sensor (tilt, impedance), etc. Although blind systems offer operational efficiency, the risk of having faulty recordings or data loss is high, thereby compromising the quality of data recorded [26]. For most seismic exploration companies, it is of significant importance to have a system that provides some QC information and transmits the seismic data acquired during acquisition, often referred to as real-time systems. A real-time system supports the instant transmission of data from equipment in the field to the CCU during acquisition.

In cable-based systems, geophones have predetermined communication channels with a fixed bandwidth to transmit seismic data for recording, as well as QC and noise level monitoring data, to the CCU in real-time. However, in cable-free systems, geophones employ dedicated infrastructures and technology to transmit data over a radio link to the CCU. In such systems, real-time data acquisition requires that data collected by geophones from a particular shot be delivered to the CCU without delaying or interrupting the next shot [12]. Although such systems are termed real-time systems, in reality, data are transmitted to the CCU with a reasonably short latency that depends on the spread or survey configuration, ranging in seconds, and are more factually characterised as “near real-time” systems [26].

## 3. Wireless Seismic Data Acquisition (WSDA)

The growing interest in large-scale seismic data acquisition for better subsurface image quality, flexibility, and automation in surveys [27], as well as impediments imposed by the cable, is some of the fundamental rationale for moving towards wireless seismic acquisition systems. Such systems often employ geophone units equipped with wireless transceivers to form a wireless geophone network (WGN) deployed over the survey area. WSDA enables a greater trace density and increased productivity, thereby increasing the earth subsurface imaging quality as compared to cable-based systems [28]. A WGN comprises of a group of wireless geophones that monitor and record ground movements, transmitting their data via radio link to another node in the network, usually a wireless gateway, towards the direction of a sink node or CCU. The gateway acts as an intermediate node and also provides coordination to a group or subnetwork of geophones in the network [29]. In the following text, we outline some requirements necessary to set up geophone networks for WSDA.

### 3.1. Network Coverage/Deployment

**Figure 3:**
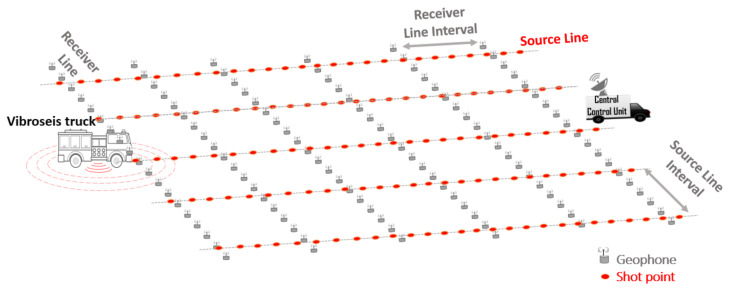
The wireless geophone network orthogonal geometry.

Depending on the survey size, a typical land seismic exploration comprises of 10,000 to 30,000 geophones [30], covering an area in the order of several square kilometres with an average density of up to 2000 devices per km ^2^. According to [9], the amount of traffic or connections in a typical survey is similar to what telecommunication operator handles in a low-populated city. Seismic surveys often employ orthogonal geometry [31] with source and receiver lines deployed perpendicular to each other according to predefined survey parameters, as shown in Figure 3. The orthogonal deployment is normally employed to combat the problem of aliasing noise (an effect that causes different signals to become indistinguishable when sampled) with low apparent velocities [31]. The **receiver line** (also called in-line) consists of geophones placed at regular intervals along the line. The source line consists of shot points marked at regular intervals along the line. **Shot points** are locations on the earth surface where the seismic waves are generated that propagate down the earth’s surface [32]. WGNs are often static networks as mobility of geophones is not required after deployment. This implies geophones can stay connected wirelessly long enough to support hierarchical networks with autonomous entities [9].

### 3.2. Network Throughput

As mentioned earlier, the rate at which data are generated by a geophone depends on the signal sampling interval and the resolution of the ADC used. For a survey with a minimum sample interval $T_{s}$ of 0.5 ms and a 24 ADC resolution, each geophone in the network will generate data at rate of 48 kbit/s. Data generated by geophones required to be transmitted to the CCU for each record or shot could be quite large. This can be quantified by an example that entails 30,000 seismic channels or geophones, with seismic signals sampled at 0.5 ms interval, 5 s geophone recording period, and a time lapse between records of 60 s. For each record, 240 kbit of data is generated per geophone, and an overall data of 7.2 Gbit (900 MB) is generated in the network. To keep up with the data throughput, at least 120 M/s bandwidth is required, as well as multiple radio links to share the bandwidth load [11]. A typical survey with up to 30,000 geophones transmitting data in real-time will require a sum throughput of 1.44 Gbit/s per shot, which will scale down to 720 M/s if a sampling interval of 1 ms is used. Support for such throughput is quite uncommon for traditional wireless sensor networks.

### 3.3. Latency, Localization, and Synchronization

Most exploration companies require that data acquired for a particular shooting phase should be observed for quality control in real-time prior to the next shooting phase. For some systems, seismic data acquired from one sample interval is required to be sent prior to the next sample interval [11] or within a reasonable amount of time (usually the time lapse between shots) with minimal latency. The network needs to be designed for continuous operation for up to several days, depending on the survey type or size. Information on geophone positioning and elevation is of vital importance for the surveyor to easily keep track of all geophones in the survey in case of failure or damage to ensure the robustness of the system. Most geophones use the Global Positioning System (GPS) for localization and timing. To avoid degradation of the subsurface image quality, accurate positioning with the error below 1 m is necessary after deployment. Seismic reflections from the earth subsurface are simultaneously detected and sampled by geophones in the vicinity of seismic sweep within the survey area. Acquisition needs to be synchronised with the time-reference distributed over the survey area and a maximum acceptable timing skew/jitter of 10–20 μs [29].

### 3.4. Channel Access

The medium access control (MAC) sublayer of the link layer is responsible for coordinating how nodes or geophones access the shared wireless medium for data transmission and reception. An effective MAC scheme will provide the best network performance by ensuring fair access to the shared radio channel (medium) and ensuring minimal collision of packets, subsequently leading to increased network throughput, low latency as well as low energy consumption [33]. In wireless sensor networks (WSN), the topology of the network plays a vital role in choosing which MAC scheme to implement. Topology in WSNs are broadly categorised into two [34], flat and cluster topologies.

In flat topology sensor nodes have the same functionality in the network and nodes can transmit data directly to the sink in single-hop or multi-hop communication without the need for central coordination. In cluster-based topologies, however, nodes are classified into cluster heads and cluster members. Cluster heads serve as a coordinator and control how cluster members access the shared wireless medium. Carrier Sense Multiple Access with Collision Avoidance (CSMA/CA), a random access scheme based on the exponential back-off algorithm is often employed in flat-based topologies as the fundamental algorithm to access the medium since no coordinator is employed [33]. In random access schemes, nodes asynchronously contend for channel access whenever they have packets to be sent. The probability of collision increases as the number of nodes or offered traffic increases. Although random access or contention-based approach provides good scalability in WSNs, some of its major drawbacks include collision, idle listening, overhearing and control packet overhead [35]. On the other hand, most cluster-based networks employ Time Division Multiple Access (TDMA) or schedule-based MAC schemes in accessing the medium [33]. In such schemes, nodes access the channel in an orderly manner. Resources (time or frequency) are pre-assigned to avoid collisions, as each node has a unique time slot to transmit its data. In the event that a node has no data to transmit, its allocated resource is wasted. To avoid underutilization of resources, schedules can often be changed by re-allocating time slots when needed [36]. However, this comes with the cost of adding overhead to protocols or schemes. In TDMA, time synchronization is required amongst nodes to avoid overlapping transmissions as well as guard slots to separate users. Although random access schemes are vulnerable to collisions, they are more scalable in terms of static channel allocation as compared to TDMA [37]. In addition, for a heavy traffic load with a short fixed time slot, the end-to-end delay will be prolonged [35].

## 4. WSDA: State of the Art

The growing demand for better subterranean image quality by exploration industries has pushed for the increase in the number of channel counts and denser acquisition, consequently leading to the migration from cable-based to cable-free acquisition systems. Any seismic acquisition system that employs a cable-less recording unit is often termed as “nodal system” [28]. The use of nodal systems is dated as far back as the 1980s. Fairfield Box and Opseis Eagle in the 1980s and 1990s are examples of systems that use the narrow-band VHF radio to send seismic data from field units to the central control unit [38]. Based on the scope and technique of quality control monitoring, the most recent nodal systems available today can be classified as follows [26]:

- **Blind recorders** are often an autonomous, battery-powered with no or extremely limited radio connectivity acquisition unit which has no means of collecting data during acquisition. Data are obtained days after the survey begins by transporting the units to the CCU and downloading their data. Timing synchronization is achieved via GPS in such units.
- **Quality control capable wireless systems** enable the collection of QC data and some seismic data by survey crew during data acquisition wirelessly. In such systems, no communication channel to the CCU is present for neither seismic data nor QC data, as such requires survey personnel to travel across the spread to collect data manually.
- **Remote quality control wireless systems** provide means of transferring operation QC data only in near real-time via a low-speed radio channel from the geophones to the CCU in the form of dynamic autonomous mesh network. Survey crews moving across the survey field can collect seismic data. Units in such systems monitor and transmit QC data as well as ambient field noise level as soon as it is detected to be out of the tolerance condition. The data rate for QC data commonly ranges from 1 to 10 B/min [9], which is of significantly lower magnitude as compared to the seismic data.
- **Real-time nodal units** transmit both seismic and QC data to the CCU directly in real-time or with minimal latency during operation. This enables contractors to monitor the quality of data acquired for a particular shooting phase before moving to the next. Such systems are similar to cable-based systems in operation and use dedicated radio links and infrastructure in their operation. Such systems use a predefine carefully planned network architecture or topology to relay a huge amount of acquired seismic data and QC data via some form of multi-hops between nodes to the CCU.

Table 1 gives a summary of some recently introduced nodal acquisition systems and their specification.

### 4.1. Review of Recent Advancement in Wireless Seismic Data Acquisition

Cable-free land seismic acquisition is discussed in several research articles [10,11,25,28,39,40,41], with deep insight into land seismic surveys and the requirements necessary to achieve optimum results in such a survey. Many others discussed wireless geophone network architectures and technologies for large-scale land seismic survey [8,9,12,29,30,42]. The most recently introduced real-time wireless system aiming at large seismic channel count is the RT3 system developed by “Wireless Seismic Inc.” (Stafford, TX, USA) in 2017 [28]. The system comprises of a recording unit called “mote”, a ground relay unit (GRU) that serves as the gateway, and a CCU called the central recording system (CRS) that supports over 250,000 seismic channels. Unlike the conventional acquisition system that follow multiple 2D line grid geometry, the RT3 system supports flexible survey geometries as well as the Hybrid Telemetry system, providing an uninterrupted seismic data acquisition in case wireless connectivity temporarily fails in some portions of the survey. The system uses the license-free 2.4 GHz ISM band for wireless communication [43].

In the RT3 system, the acquisition units and gateways are controlled and managed by the CCU. Each recording unit transmits its data to the GRU associated with it using a Time Division Multiple Access (TDMA)-based MAC protocol. The relay unit then forwards the data from one GRU to another in a multihop manner until it reaches the CRS, where the seismic data acquired is processed. The GRU is a full duplex transceiver, which significantly reduces communication latency and allows the RT3 to scale over 250,000 channels with full real-time data transmission. The sustained throughput of the GRU sub-system is approximately 20 Mbit/s with a “burst” rate of up to 55 Mbit/s. The GRU radio network is a fully automated formation sub-system, such that the GRUs self-organise with adjacent GRUs. A GRU is typically mounted on a tripod and, depending on the terrain and user-selected height, the distance separating the GRUs is usually between 100 and 400 m [43].

### 4.2. Technologies for Wireless Geophone Networks

There is plenty of research that discuss technologies for wireless seismic data acquisition networks. Authors in [12] discussed the feasibility of employing WiFi, WiMax, Bluetooth, ZigBee, and Ultra-Wideband (UWB) technologies in WGN. However, considering the WGN requirements in terms of the data rate, network size (in case of Bluetooth), power consumption, real-time data transfer, etc., these technologies might not be the best options. An elaborate explanation of employing UWB technology in WGNs is discussed in [9]. The authors proposed UWB technology as a reasonable choice at the physical layer considering the advantage of large bandwidth (greater than 500 MHz) to provide high-quality delay and a high data rate to support bursty traffic. However, due to a short communication range (5–10 m for up to 480 Mbit/s, and 30 m expected for outdoor line-of-sight environment with 53.3 Mbit/s data rate [9]) confined in UWB, geophones will be operating relatively near their range limit with a multi-hop form of communication to convey seismic data. The design of a wirelessly distributed three-component seismic data acquisition system based on Long Range (LoRa) wireless technology is discussed in [44]. The system monitors the parameters of seismic waveform, such as sampling rate and signal amplification, in real-time during the survey. The data collected by the system are locally stored on an SD card and then forwarded to the monitoring unit via LoRa technology for further processing. However, LoRa might not be the best candidate for WSDA, especially considering data rate requirements in high-density WGNs. In addition, the authors in [45] proposed an Internet-of-Things-based Low-Power Wide-Area (LPWA) WGNs with a focus on seismic quality control QC data not a real-time WSDA system. In [20], the authors proposed a wireless geophone network based on the IEEE 802.11af standard that enables WLAN operation in television white space (TVWS), which can achieve longer communication range, providing more network scalability in WSDA. IEEE 802.11af occupies many various pre-licensed bands TVWS channels, as such its operation might be limited based on potential regional interference and access regulations [46]. An integrated energy-efficient wireless sensor node for microtremor survey method was proposed by the authors in [47]. They suggested a data quality monitoring system that solves some technical issues between the recording unit and the control centre through 4G wireless monitoring technologies. Authors in [48] proposed a wireless exploration system based on a mixture of WLAN and LTE technology. Although LTE can offer considerable data rates, spectrum licensing and deployment costs could be a cause for concern.

Table 2 gives the summary of the technologies likely to serve as the main solution in the wireless seismic data acquisition for both long and short range communication [12].

In general, considering the large survey field layout and geophone density in WGNs, the selection of wireless technology for a reliable and efficient data acquisition should take into account the communication range and high data rate, as well as imposed costs and power consumption. Obtaining a single wireless communication technology that addresses all these can be quite difficult. Proposals for a mixture of short and long range technologies have been put forward in literature. Nonetheless, employing short range technologies with the multi-hop form of communication to relay seismic data from geophones to gateways down to the CCU could be an alternative approach. However, appropriate routing protocols need to be defined in such scenarios.

### 4.3. Network Architecture

Conventional orthogonal geometry where receiver lines and source lines are placed perpendicular to each other is often employed in seismic surveys. A receiver line interval and an inter-geophone distance are pre-defined according to the survey parameters before the commencement of data acquisition. A denser receiver arrangement leads to better subsurface image quality. Savvazi et al. in [9] proposed a hierarchical WGN architecture in which the survey area is broken down into subnetworks, with each subnetwork managed and coordinated by a wireless gateway. Subnetworks are further organised into clusters where geophones connect to the associated cluster heads and transmit their data to. The cluster heads then relay the aggregated data from geophone members to the gateway using short range UWB communication technology and from the gateway to the CCU using long range WiFi technology. However, no performance evaluation was carried out in the proposed architecture. Furthermore, due to the short communication range in UWB technology, a multi-hop network is needed to convey data from geophones, which might be operating near their communication range limit at best. Moreover, most of the data traffic is expected to be relayed via the cluster heads closest to the gateway, raising concern for coordination and energy consumption in such nodes.

An architecture that defines a serial data transfer path amongst geophones is described in [49]. Geophones are deployed in series to relay seismic data in a chain-like pattern to the CCU using multiplexing techniques, such as time or frequency division multiplexing. Although the spectrum is effectively utilised, data acquisition time might be high, as well as power consumption of nodes. A wireless architecture based on the IEEE 802.11af standard is proposed in [20]. In this architecture, the survey area is divided into hexagonal cells based on the frequency reuse concept, such as in cellular communication. The cluster size is determined by the number of available TV white space channels, and each cell consists of a wireless gateway that collects data from geophones within the cell area in a star topology. However, issues of co-channel interference, geophone power consumption as well as regional regulations for accessing TV white space spectrum may be a cause for concern. In the RT3 system of wireless seismic [43], multiple relay units (gateways) are employed in the network, where a group of geophones (referred to as motes) for a portion of the survey will be associated with and will transmit their data directly to this relay unit. Each relay unit will then relay the acquired data to the CCU wirelessly, either directly or through multi-hop communication with neighbouring gateways. Authors in [48] proposed a dual-layer network (lower and upper layer network) design for the WSDA system. The lower layer employs a star network topology comprising of eight wireless geophones and a gateway to form a subnetwork, communicating based on 802.11 WLAN technology. In the upper layer network, the gateway in each subnetwork forwards the aggregated data to the CCU via the LTE network. However, a significant amount of gateways will be required with such network architecture.

Generally, the architecture in WGN should be scalable to a high number of nodes. In addition, it should also be able to address support for real-time data transmission, data reliability, and low latency.

## 5. Proposed Network Architecture/Acquisition Technique

**Figure 4:**
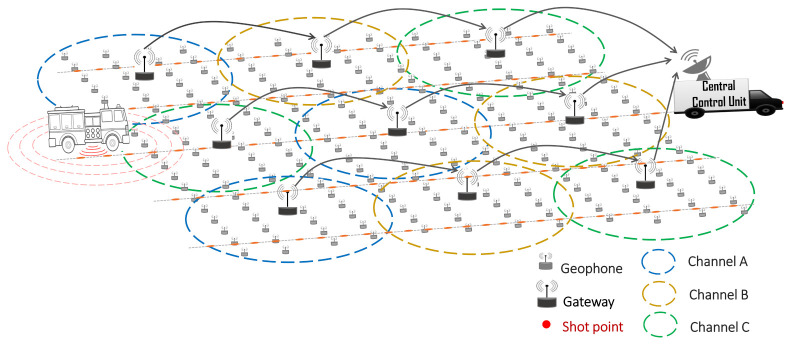
The proposed WGN architecture.

To investigate the feasibility of future WSDA applications using the traditional approach and to identify bottlenecks and problems, we proposed a WGN architecture and an acquisition technique. Similar to [48], the survey area is divided into subnetworks, as shown in Figure 4. Each subnetwork is managed by a wireless gateway that serves as a central coordinator and data aggregator for a number of geophones that fall within its communication range. Each geophone in the subnetwork transmits its sensed data to the gateway via 802.11 WLAN technology. To limit the effect of adjacent and co-channel interference, neighbouring gateways are expected to use non-overlapping channels (for example, the three non-overlapping channels in the 2.4 GHz band (channels 1, 6 and 11)), in such a way that no adjacent gateway in a subnetwork uses the same channel. These channels can then be reused in different subnetworks throughout the survey area. The gateway then relays the aggregated data to the CCU via multi-hop communication with neighbouring gateways or via single-hop communication using a longer range communication technology. Data compression techniques could also be applied at the gateways to reduce the high bandwidth requirement or the number of bits transmitted to the CCU. This proposed architecture approach could be quite promising in terms of scalability and better network planning and management. The gateways should have a dual interface to be able to support both short and long range wireless technologies.

**Figure 5:**
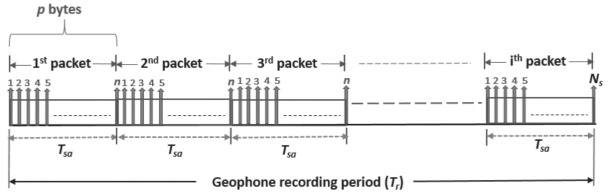
Geophone recording period acquisition data traffic generation.

However, in this work, we focus on investigating the performance of the proposed acquisition technique for a given number of geophones in a single subnetwork. As most seismic acquisition applications require data to be transmitted after the geophone recording or listen period ($T_{r}$), in this work, we propose data be transmitted amid $T_{r}$, referred to as “recording period acquisition”. This provides a form of real-time data transfer, thereby significantly increasing the overall seismic survey productivity. A number of seismic data samples (*n*) collected by geophones over a given period of time ($T_{sa}$) during $T_{r}$ for a particular sweep is transmitted to the CCU at the end of every $T_{sa}$ period prior to the next $T_{sa}$ until the end of $T_{r}$, as depicted in Figure 5. $T_{sa}$ represents the seismic sample accumulation time and defines how frequent packets are sent by geophones in the network. In this work, a typical value of $T_{r}=5\;s$ is used.

Imagine a survey in which $T_{sa}=1\;s$ is defined. This means each geophone will acquire a number of seismic data samples over the period of 1 s, and the corresponding data samples will be placed in a packet and sent over the network prior to the next $T_{sa}$ until the end of the 5 s recording period $T_{r}$. In this example, a total of five packets will be generated per geophone for the duration $T_{R}=5\;s$. However, this also means that 5 times as much channel access will be required as compared to 1 time channel access with one larger packet containing all seismic data samples accumulated over the period $T_{r}=5\;s$. Authors in [20] have discussed a data collection scheme that can be applied during the listen interval using a TDMA-based protocol, investigating the optimal time slot to be allocated to geophones during listen interval.

In our work, each geophone is assumed to have a 24 bit ADC resolution and sample interval of 0.5 ms. With this, the response of a single geophone will generate $N_{s}=10000$ seismic data samples for a single sweep, with each sample having a data size of 3 B. For the recording period acquisition, we investigate for an optimal number of seismic data samples “*n*” (a function of the packet payload size “*p*”) that could be sent during the geophone recording period and how often this data samples should be transmitted over the network. The relationship between *n* and *p* is given in Equation (4).

### 5.1. Scenario

**Figure 6:**
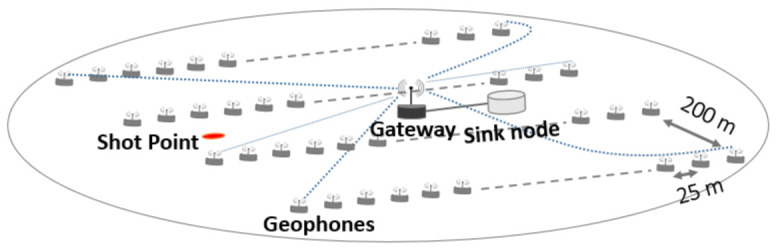
Scenario.

As the proposed network architecture consists of a number of subnetworks, we focus on investigating the geophone recording period acquisition technique for a single subnetwork only with a defined number of geophones to obtain an initial insight into the network’s performance. Figure 6 depicts the scenario under which the study was carried out. A single-fleet operation with one vibroseis truck, and single shot was assumed. A subnetwork from the network architecture proposed in Figure 4, consisting of 100 geophones within the communication range of a gateway, was considered. The gateway acts as an access point or relay unit to a sink node, where geophones transmit their seismic data to, as well as provides coordination to the geophones, as defined in the IEEE 802.11 Basic Service Set architecture. The subnetwork consists of four receiver lines placed at intervals of 200 m. Each receiver line consists of 25 geophones separated by a distance of 25 m and spread over an area of 0.36 km. The gateway is placed at the centre and connected to the sink node via a high speed cable (100 Gbit ethernet). This is employed to model an ideal connection between the gateway and the sink node in the simulation, as our scenario considers one subnetwork only and the gateway does not relay data to the CCU.

We examine an optimal *n* in terms of payload size *p* to be sent over the network, which will ensure the best network performance in terms of packet loss and latency, employing the IEEE 802.11 g technology in relation to the defined wireless seismic data acquisition scenario, such that

$$1⩽n<N_{s}\;\mathrm{or}\;3⩽p<P$$

where $N_{s}$ is the total number of seismic samples generated per geophone for the period of time $T_{r}$ (10,000 samples), and *P* is the maximum data payload size generated per geophone for the period $T_{r}$ (30 kB). *p* can be expressed in terms of *n* using the relation in Equation (4).

(4) p=n×3B

For real-time seismic data acquisition, it is required that data acquired from one sample interval are transmitted prior to the next sample interval [11] or within a reasonable amount of time (usually the time lapse between shots) with minimal latency. A delay bound of 1 s if defined as a measure of the QoS in our WGN scenario, within which it is expected that all data from geophones in the network are received at the sink node after the period of time $T_{r}=5\;s$, and the acquired seismic data QC check can be performed for that shot before moving to the next shot point. This mean that we defined a total acquisition time with a lower bound equal to 5 s and an upper bound of 6 s (5 s listen period plus 1 s). An additional measure of QoS considered is that there should be 0% packet loss in the network.

### 5.2. Geophone Data Traffic Generation

Figure 5 illustrates the data traffic generation pattern of the geophone recording period acquisition. With each geophone having a 24 bit ADC resolution and a sampling interval of 0.5 ms, the reflected seismic signal will be sampled at a rate of 2000 samples/s, with each sample having a data size of 3 B. Data will be generated at a rate of 6 kB/s (48 kbit/s) per geophone. For the 5 s recording period, the data generated per geophone ($D_{g}$) is 30 kB (240 kbit). For any given value of *n*, the payload size *p* and $T_{sa}$ can be computed using Equation (5) and Equation (6), respectively.

(5) $$p=\frac{n\times D_{g}}{N_{s}}$$

(6) $$T_{\mathrm{sa}}=\frac{n\times T_{r}}{N_{s}}$$

$T_{sa}$ can also be expressed in terms of *p* and $D_{g}$, as given in Equation (7).

(7) $$T_{\mathrm{sa}}=\frac{p\times T_{r}}{D_{g}}$$

The theoretical offered load (η) in the network for different payload sizes can be computed using the relation expressed in Equation (8).

(8) $$\eta =\frac{8\times p\times N\times N_{ps}}{T_{x}}\;\mathrm{bit}/s$$

where *N* denotes the number of geophones in the network, *p* is the payload size, $N_{ps}$ is the number of packets sent per node within 5 s recording period in bytes, and $T_{x}$ is the time taken to receive all data packets at the sink, given by ($T_{r}-T_{sa}$). $N_{ps}$ can be computed from Equation (7) using the expression below:

(9) $$N_{ps}=\frac{D_{g}}{p}=\frac{T_{r}}{T_{sa}}$$

### 5.3. Wireless Technology

Owing to its ubiquitous and license-free nature along with modelling basis, the IEEE 802.11 g technology that operates in the 2.4 GHz ISM band is employed. It has a maximum data rate of 54 Mbit/s and utilizes a 22 MHz channel bandwidth, making it possible to indulge three non-overlapping channels in the 2.4 GHz ISM band. The 802.11 medium access control (MAC) sublayer architecture defines channel access mechanisms that includes the Distributed Coordination Function (DCF), the optional Point Coordination Function (PCF), and Hybrid Coordination Function (HCF) [50]. DCF is the fundamental access mechanism in 802.11 in which stations (nodes) contend for access to the medium in a random manner based on the carrier sense multiple access with collision avoidance (CSMA/CA) protocol, where collisions are managed according to binary exponential back-off rules. DCF defines two techniques for packet transmission: the basic access and the request-to-send/clear-to-send (RTS/CTS) mechanisms. Basic access mechanism is the default DCF scheme in which, following a successful reception of packet by the destination station, an acknowledgement is immediately transmitted to the source station. In the RTS/CTS scheme, the source station sends a RTS frame to reserve the channel before transmitting a packet. A CTS frame is sent back by the destination to the source station to acknowledge the receipt of the RTS frame. Subsequently, the normal packet transmission and acknowledgement response occurs. In this work, we limit our investigation to the DCF basic channel access mechanism. The RTS/CTS access mechanism is turned off.

## 6. Performance Analysis

In this section, we evaluate the network performance of the proposed acquisition scheme for different seismic sample or data payload sizes and sample accumulation time for a single seismic sweep, as depicted in Figure 5.

### 6.1. Performance Metrics

While evaluating the network performance, we focused on four metrics or performance indicators. This includes throughput, average end-to-end delay, packet loss due to retransmission failure, and the number of retransmitted packets.

#### 6.1.1. Throughput

Defines the amount of data successfully received from geophones at the sink node within a given period of time. For *K* number of packets, each of size *D* bit, we express the throughput as:

(10) $$\frac{KD}{T}\;\mathrm{bit}/s$$

where *T* is the total time required to transmit all data packets from geophones to the sink node, also given as the difference between the timestamp when the last and first packets are received.

#### 6.1.2. Average End-to-End Delay

Measures the average time it takes to transmit data packets from all geophones in the network to the sink node. This includes packet transmission, propagation, queueing, and processing time. It is expressed as:

(11) $$\frac{1}{K}\sum_{i=1}^{K}\left(T_{i}^{r}-T_{i}^{s}\right)$$

where $T_{i}^{s}$ is the instant the *i*th packet is generated and sent out by a geophone, $T_{i}^{r}$ is the time the *i*th packet is received at the sink node, and *K* is the total number of packets received at the sink node from all geophones.

#### 6.1.3. Packet Loss Due to Retransmission Failure Ratio

This metric measures the amount of dropped packets by a geophone at the link layer due to failed retransmission. This occurs as a result of exceeding the retransmission attempt limit due to multiple collisions. It is expressed as:

(12) $$\frac{\sum_{i=1}^{N}p_{d_{i}}}{\sum_{i=1}^{N}p_{u_{i}}}$$

where $p_{d_{i}}$ is the number of packets dropped by the *i*th geophone due to retransmission failure; $p_{u_{i}}$ is the number of packets sent from upper layer to the link layer of the *i*th geophone; *N* is the total number of geophones.

#### 6.1.4. Retransmission Ratio

This metric shows the percentage of packets sent with retransmission by geophones in the network. It is defined as the ratio of number of retransmitted packets per geophone ($p_{r}$) (as a result of been lost or failure to reach the destination during their initial transmission) to the total number of successfully transmitted packets ($p_{s}$) by the geophones to the sink node during both initial transmission and retransmission attempt.

(13) $$\frac{\sum_{i=1}^{N}p_{r_{i}}}{\sum_{i=1}^{N}p_{s_{i}}}$$

### 6.2. Simulation

The OMNeT++ discrete event simulator is used [51]. Table 3 outlines the simulation parameters used. A flat ground open area environment, as well as free space path loss model, is considered. The simulation was carried out for 10 runs. As the sink node is connected via Ethernet, which has a maximum transmission unit (MTU) size of 1500 to the access point, we configure a WLAN MTU size of 1500 in the simulator to avoid the fragmentation of packets.

### 6.3. Simulation Results

**Figure 7:**
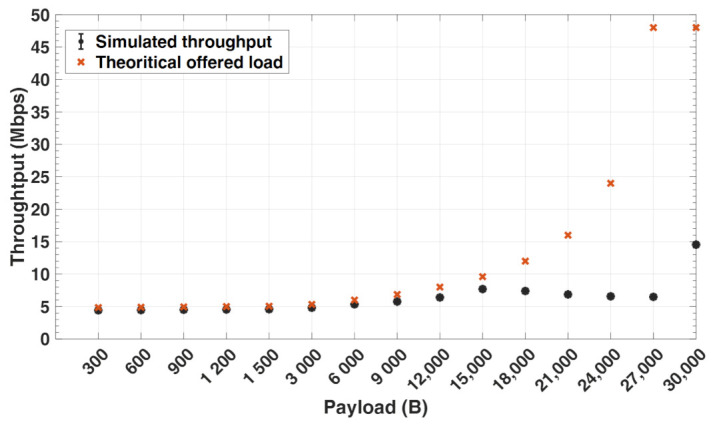
Application level throughput vs. offered load.

In this section, we evaluate some of the WGN key performance metrics from the simulation for different payload sizes *p*. Figure 7 shows the plots for the offered load to the network as compared to the throughput measured in the simulation. The offered load is computed analytically using Equation (8). It can be observed from the figure that the offered load increases with increase in payload size. This is because the sample accumulation duration ($T_{sa}$) increases with a higher payload size, consequently reducing the time it takes to receive all data packets at the sink node ($T_{x}$) over the 5 s geophone recording period duration ($T_{r}$). The lower the $T_{x}$, the higher the offered load. As $T_{x}$ for both packet size 27 kB and 30 kB are nearly the same, the offered load for both packets sizes is almost the same, as can be seen from the figure. Furthermore, with larger packet size, this would mean more available data in the network and also higher throughput, as long as the capacity is not exceeded.

**Figure 8:**
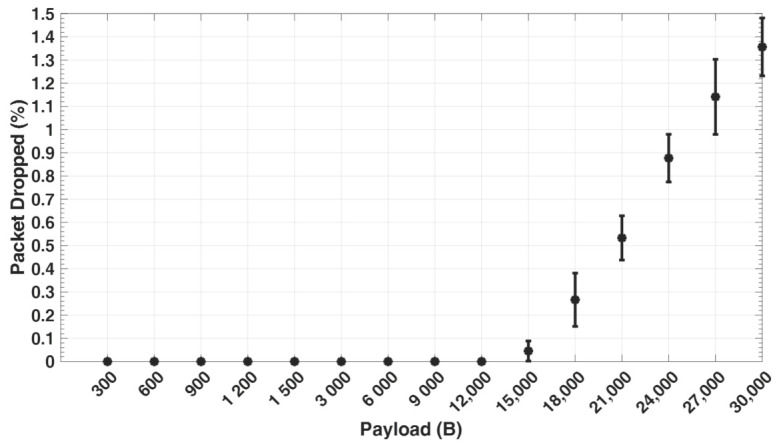
Packet loss due to retransmission failure.

In comparison with the measured throughput from simulation, it can be seen that the throughput closely follows the offered load up to 15 kB payload size. Beyond 15 kB, the throughput starts to drop significantly. This may be explained by the fact that packets get fragmented at the network layer once the payload exceeds the MTU size, resulting in additional smaller packets (fragments). Each of the fragmented packets have their own header, which results in an increase in overhead and consequently a decrease in throughput. Furthermore, each of the fragmented packets contend to access the wireless channel, which consequently increases network congestion at the link layer. After several retransmission attempts by nodes, some of the packets are dropped, owing to the fact that the retransmission attempt limit is reached, as can be seen in Figure 8. Throughput at 30 kB payload, which represents the entire seismic data recorded for the duration $\left(T_{r}=5\;s\right)$, tends to be significantly higher. This is because each geophone generates and transmits 30 kB UDP packet only once over the network, which is not the case for lower packet sizes, but gets fragmented into smaller packets at the network layer.

**Figure 9:**
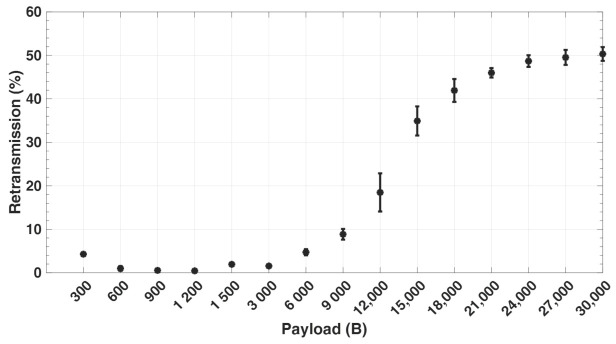
The packet retransmission ratio.

**Figure 10:**
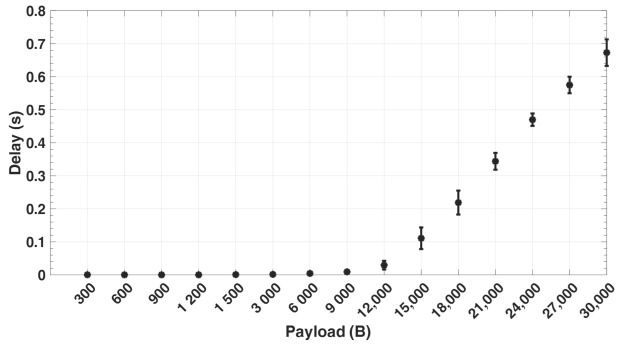
The average End-to-End Delay.

Figure 9 shows the percentage of packets sent out with retransmissions by geophones for different payload sizes, as captured from the simulation. The greater the number of packets at the link layer, the higher the retransmission ratio. It can be noticed that the retransmission ratio at 300 and 600 payload size is higher than at 900 and 1200 payload size. This is because with a lower payload size, more packets ($N_{ps}$) are sent by each geophone with a smaller send interval ($T_{sa}$) within the period ($T_{r}$), thereby resulting in more congestion at the link layer and hence more collisions. With larger payload sizes, the percentage of retransmissions significantly increases due to the increase in the number of fragmented packets. For every retransmission, this implies that additional packet transmission, propagation, queuing, and processing time is required, which consequently leads to an increase in the overall end-to-end delay. This can be seen in Figure 10, which captures the average end-to-end delay for different payload sizes in the network from the simulation. Moreover, a packet arriving at the sink node is successfully received at the application layer only when all of its fragments are correctly received, thereby increasing the delay.

## 7. Discussions

An initial objective of this study was to determine an optimal value of *n* (a function of *p*) that could be sent during the geophone recording period that ensures the best network performance in accordance with the defined QoS in our WGN setting. The results of this study indicate that the throughput can be maximised by sending the largest possible value of seismic data sample size *n* or payload *p* over the network. This, however, comes with a price of fragmentation at the network layer due to the limit on the WLAN maximum transmission unit (MTU) size (1500 B). The larger the value of *n*, the higher the load on the channel, thereby increasing the chances of packets being dropped, leading to decrease in the throughput. In addition, this will also lead to a greater number of retransmissions, thereby degrading the overall network performance in terms of delay and packet loss. To determine the optimal value of *n*, a comparison between throughput, delay, and packets dropped from the results obtained was carried out in relation to our predefined QoS parameters in this study (1 s upper bound delay and 0% packet loss). Based on this, we defined n=4000 (which corresponds to data payload size of 12 kB) as optimal because it has negligible packets dropped, higher throughput compared to smaller values of *n*, and also delay falls within our defined bounds. However, it is important to note that this study has a number of limitations. Firstly, a WLAN MTU size of 1500 was considered, which implies that the optimal value of *n* might vary for different MTU sizes. Secondly, we assumed a free space path loss propagation model. Switching to a more realistic channel model such as the two-ray propagation model will deem more appropriate as geophones often have antenna heights of less than 1 m. Furthermore, the study was carried out with only 100 geophone nodes and one gateway for a single subnetwork under ideal channel conditions. Reducing the number of geophones in a subnetwork will, however, increase the optimal value of *n* (or *p*) as a result of the reduced contention at the MAC layer. Nevertheless, the objective is to utilize the maximum number of geophones within a subnetwork, which gives the expected network performance, thereby reducing the overall number of gateways used in the survey. Future work will take the above limitations into account.

## 8. Conclusions

In this paper, we presented a general overview on land seismic data acquisition. A summary of some fundamental requirements necessary to set up a network of wireless geophones in a seismic setting was also outlined. Furthermore, a review on state-of-the-art wireless seismic survey systems and enabling technologies for such networks was summarised. The proposed wireless geophone recording period acquisition in this study was investigated for a subnetwork of a WSDA network setting and evaluated in the OMNeT++ simulator. Based on the simulation results obtained, it is clear that an optimal number of seismic samples *n* to be sent by each node during the recording period depends on the MTU size in the network and QoS service parameters as pre-defined in any particular seismic survey (e.g., delay bounds). More frames at the MAC layer resulting from network layer fragmentation due to larger payload size or higher load on the channel leads to congestion and higher collision probability, which subsequently degrades the WGN performance. Furthermore, the study shows that transmitting data at the end of geophone recording period might lead to a higher probability of packet loss in the network. Based on the acquisition scenario in this work, n=4000 (equivalent to packet size of 12 kB) is optimal.

### Future Work

Future work will be directed towards investigating for more than 100 geophones to define an optimal number of geophones that could be supported in each subnetwork for the proposed WGN architecture. Analytical modelling for some key performance indicators, such as throughput and collision probability, will also be looked into. Furthermore, we will investigate the WGN performance for different MAC protocols and wireless communication technologies. With longer range technologies, such as 802.11ah, 802.11af, or LTE, more geophones can be covered or coordinated by a single gateway, which will consequently minimize the number of gateways used in the survey. However, with shorter range communication technologies, a smaller number of geophones in a subnetwork will be associated to the gateway, thereby resulting in a significantly high number of gateways to be deployed in the entire seismic survey. An approach to remedy this will be the use of multi-hop communication between geophones to relay seismic data to the gateway and gateways to the CCU. In the near future, we will investigate an effective routing protocol to be employed in wireless seismic data acquisition.

## Figures and Tables

**Table 1:** Recent nodal seismic units and their specifications.

Device	Operating Mode	Battery Operating Life Time	Storage/Technology Employed	Sampling Interval/ADC Resolution	Manufacturer/Website
Innoseis Tremornet	Blind	50 days continuous	Low energy bluetooth	1, 2, 4 ms/ 24 bit	Innoseis/www.innoseis.com
GCL Connectorless recorder	Blind	60 days @ 24 h/day	16 or 32 GB storage	0.25, 0.5, 1, 2, 4 ms/ 24 bit	Geospace Technologies/www.geospace.com
DTCC smart solo	Blind	25 days @ 24 h/day	8 GB memory	1, 2, 4 ms/ 24 bit	SmartSolo/www.smartsolo.com
GTI NRU 1C	Blind	23 days @ 24 h/day	8 GB memory	0.5, 1, 2, 4 ms/ 24 bit	Geophysical Technology/www.geophysicaltechnology.com
Sercel WTU 508	Real-time QC	30 days @ 24 h/day	WLAN (a, b, g, n),XT-Pathfinder	0.5, 1, 2, 4 ms/ 24 bit	Sercel/www.sercel.com
RT2 system	Real-time	25 days @ 12 h/day	WiFi 2.4 GHz ISM band	0.5, 1, 2, 4 ms/ 24 bit	WirelessSeismic/www.wirelessseismic.com
RT3 system	Real-time	27 days/battery charge	WiFi 2.4 GHz ISM band	0.5, 1, 2, 4 ms/ 24 bit	WirelessSeismic/www.wirelessseismic.com

**Table 2:** Technologies for wireless seismic survey [12].

Technology	Comm. Range (m)/Type	Data Rate (Mbit/s)	Power Consumption mJ/MB
ZigBee	50/Short Range (SR)	0.25	8–32
MB-OFDM	10/SR 30/SR	200 53	12–16 8
UWB-Impulse Radio (UWB-IR)	50/SR	1–5	8
GSM (EDGE)	1000–2000/Long Range (LR)	0.384	300 J/M
3G (UMTS)	1000–2000/LR	2	80 J/M
4G (LTE)	>1000/LR	100–1000	80 J/M
5G	∼250/SR	>1000	80 J/M
IEEE 802.11g	160/SR 70/SR	6 54	120–160
IEEE 802.11n IEEE 802.11af IEEE 802.11ah	750/LR 1000–2000/LR 1000/LR	125 26.7 40	8 J/M 8 J/M 8 J/M

**Table 3:** Simulation Parameters.

Parameter	Value
Operating Frequency	2.4 G Hz
Recording Period Tr	5 s
Simulation Area	625 m × 600 m
Simulation Time	15 s
WLAN MTU	1500
Physical Environment	Flat ground
Propagation Model	Free space path loss
Background Noise	Isotropic scalar
Node Max. Transmit Power	20 dBm
Bandwidth	20 MHz
Traffic Type	Periodic (UDP based)
PHY Bit Rate	54 Mbit/s
Packet retransmission limit	7
Beacon Interval	100 ms
SIFS	10 μs
slotTime	20 μs
DIFS	50 μs

## Data Availability

The data presented in this study are available on request from the corresponding author. The data are not publicly available due to privacy reason.

## Abbreviations

The following abbreviations are used in this manuscript:

CCU	Central Control Unit
WSDA	Wireless Seismic Data Acquisition
WGN	Wireless Geophone Network
ADC	Analogue to Digital Converter
MTU	Maximum Transmission Unit
